# Ultra-Brief Breath Counting (Mindfulness) Training Abolishes Negative Affect–Induced Alcohol Motivation in Hazardous Community Drinkers

**DOI:** 10.1007/s12671-024-02315-8

**Published:** 2024-02-27

**Authors:** Alexandra Elissavet Bakou, Lorna Hardy, Ruichong Shuai, Kim Wright, Lee Hogarth

**Affiliations:** School of Psychology, University of Exeter, Washington Singer Building, Perry Road, Exeter, EX4 4QG UK

**Keywords:** Stress, Mood Induction, Mindfulness, Breath Counting, Alcohol Craving

## Abstract

**Objectives:**

Mindfulness therapy improves drinking outcomes arguably by attenuating negative mood–induced drinking, but this mechanism has not been demonstrated in hazardous community drinkers. To address this, three studies tested whether a key ingredient of mindfulness, breath counting, would attenuate the increase in motivation for alcohol produced by experimentally induced negative mood, in hazardous community drinkers.

**Method:**

In three studies, hazardous community drinkers were randomized to receive either a 6-min breath counting training or listen to a recited extract from a popular science book, before all participants received a negative mood induction. Motivation for alcohol was measured before and after listening to either the breath counting training or the control audio files, with a craving questionnaire in two online studies (*n* = 122 and *n* = 111), or an alcohol versus food picture choice task in a pub context in one in-person study (*n* = 62).

**Results:**

In Study 1, breath counting reduced alcohol craving. However, since the mood induction protocol did not increase craving, the effect of breath counting in reversing such increase could not be demonstrated. Online breath counting eliminated the increase in alcohol craving induced by negative mood (Study 2) and eliminated the stress-induced increase in alcohol picture choice in the pub environment (Study 3).

**Conclusions:**

Briefly trained breath counting attenuated negative mood–induced alcohol motivation in hazardous community drinkers. These results suggest that breath counting is a reliable and practical method for reducing the impact of negative emotional triggers on alcohol motivation.

**Preregistration:**

These studies are not preregistered.

Negative affective motivation of goal-directed drug-seeking appears to be a primary mechanism underpinning addiction (Hogarth, 2020). Longitudinal studies show that anxiety and depression, and self-reported use of alcohol to cope with negative affect predict the development and maintenance of alcohol dependence and propensity to relapse (Crum et al., 2013a, 2013b). Experimental induction of negative states (e.g., stress, sadness, pain) increases alcohol motivation (Bresin et al., 2018), and such induction effects are more pronounced in individuals with psychiatric symptoms (anxiety, depression), and in those who report substance use to cope with negative affect (Hogarth & Field, 2020), or who are prone to relapse (Higley et al., 2011; Sinha et al., 2011). Consequently, attenuating negative affect–motivated alcohol-seeking is a key target for therapy.

One objective of mindfulness-based interventions (MBIs) for alcohol dependence is to build resilience to negative affect drinking triggers (Marlatt & Donovan, 2005; Stasiewicz et al., 2018). MBIs are effective in reducing both negative affect and problematic drinking (Bowen et al., 2014; Sancho et al., 2018). Furthermore, MBIs have been shown to weaken the association between negative affect and alcohol problems, suggesting resilience to negative affect drinking triggers might mediate its therapeutic effect (Hsu et al., 2013; Witkiewitz & Bowen, 2010; Witkiewitz et al., 2013).

Experimental mood induction studies also support this claim. Such studies demonstrate that MBIs can attenuate both the emotional (Basso et al., 2019; Carpenter et al., 2019) and the craving responses to negative mood induction. For example, Carroll and Lustyk (2018) found that 8 weeks of mindfulness-based relapse prevention training in treatment-seeking drinkers abolished the effect of an arithmetic stressor on alcohol craving, compared to a treatment as usual group. Kober et al. (2017) found that a seven-session mindfulness training package (focusing on resilience to craving), in smokers of ≥ 10 cigarettes per day, attenuated neural reactivity (in the amygdala and insula) to individualized stressful/negative scripts, compared to a cognitive behavioural intervention. This reduction in neural reactivity predicted greater smoking cessation at follow-up. However, these therapeutic effects stand in contrast to four studies which have reported no effect of mindfulness therapy (Brewer et al., 2009) or ultra-brief mindfulness training (Adams et al., 2012; Luberto & McLeish, 2018; Vinci et al., 2014) on mood-induced craving. Consequently, it remains unclear what the boundary conditions are for this therapeutic effect.

A recent study from our lab tested whether training 192 student drinkers in one component of mindfulness—breath counting—would attenuate stress-induced alcohol choice (Shuai, Bakou, Hardy, & Hogarth, 2020). Breath counting is a key component in MBIs and has been previously validated as a behavioural measure of mindfulness (Levinson et al., 2014). In our recent study, baseline choice of alcohol versus food pictures was measured in two-alternative choice trials, as this behavioural assay has been well validated as an index of current relative drug value (Hogarth & Field, 2020) which can be translated to animal models (Banks & Negus, 2017). Half of participants were trained via a 6-min audio file on breath counting (Levinson et al., 2014; Wong et al., 2018), whereas the other half of participants listened to a recited extract from a popular science book. All participants were then stressed using a continuous, loud, and unpleasant industrial noise (Cherek, 1985), and alcohol picture choice was measured again, as at baseline. It was found that whereas the control group showed a stress-induced increase in alcohol choice, this effect was attenuated in the breath counting group. Breath counting also attenuated the worsening of subjective mood produced by stress induction. Thus, deployment of a briefly trained breath counting technique attenuated stress-induced alcohol motivation and subjective negative affect. The main problem with this analysis, however, was that there was a moderation effect wherein more hazardous drinkers were less susceptible to the effects of breath counting on stress-induced alcohol choice, suggesting limited efficacy for more severe drinkers.

The purpose of the three studies reported here was to test whether brief breath counting training would attenuate negative mood–induced alcohol motivation in hazardous community drinkers. Studies 1 and 2 tested this effect online, to evaluate the efficacy of electronic delivery of the brief intervention. Study 3 tested the effect in hazardous daytime drinkers tested in a pub context, to evaluate efficacy in a natural environment. The finding that breath counting attenuates negative mood–induced alcohol motivation in all three studies would demonstrate the reliability of the effect in hazardous community drinkers, and support the claim that mindfulness therapy might improve drinking outcomes via this mechanism.

## Study 1

### Method

#### Participants

Participants were community members recruited online. Inclusion criteria were individuals had to be located in the UK, they had to be native English speakers, and aged between 18 and 65. Participants also had to provide full responses to the survey. In addition, to be included in the study, participants had to have consumed at least one drink in the last 30 days, and score greater than or equal to 8 on the Alcohol Use Disorders Identification Test (AUDIT; Babor et al., 2001) indicating hazardous drinking (see below). Exclusion criteria were reporting drug use on the day of the experiment, outlying long survey times (more than 3 hr as in Buchanan & Scofield, 2018), or aberrant responses to open-ended questions. Six open questions asked participants to write their favourite candy, dessert, snack, fizzy drink, and sporting event and sport to detect automated fraudulent responders (Pratt-Chapman, 2021). Responders with nonsensical responses were excluded. Two hundred ten participants met the inclusion criteria and of these, 73 were excluded for providing aberrant responses to open-ended questions or long survey times, and 15 were excluded because they reported drug use on the day of the study leaving 122 participants (52.4% male) in the final analysis, 68 in the breath counting group, and 54 in the control group. Although our final sample size was reduced from 210, the final sample size remains powerful at > 99% to detect a negative mood induction effect.

All participants provided informed signed consent and those with complete responses plus no nonsensical responses to open-ended questions received £3 Amazon vouchers as a reimbursement. The study was approved by the School of Psychology Research Ethics Committee.

#### Procedure

Using simple random sampling on a 1:1 ratio, participants who met the inclusion criteria were randomly assigned to the breath counting or control intervention. The breath counting group received the following instructions: “*You will now listen to a 6-min audio file and learn a meditation technique that has been practised for thousands of years. After the audio file is played, a small arrow will appear on the screen that will allow you to move on to the next step. We recommend using headphones for this exercise. Please make sure that you have set the audio volume to a comfortable level. Please be aware that for the length of this audio file, you will not be able to click anywhere else on this web page. Try to pay careful attention to the recording, and avoid doing anything else at the same time*.” Participants then listened to a 6-min audio file (Ramsburg & Youmans, 2014) available online: https://www.youtube.com/watch?v=tnFUvLIBhKQ) in which they were instructed (via a female voice) to relax and concentrate on their breath sensations, then count each outbreath, at normal pace, from one to ten, and then start again from one. The control group received similar instructions and listened to a 6-min audio file which recited (in the same female voice) an extract from the popular science book “A Short History of Nearly Everything” by Bill Bryson (Shuai et al., 2020). Breath counting was expected to attenuate reactivity to mood induction that followed.

All participants then received the negative mood induction procedure. Participants were asked to watch a 3.5-min video containing 32 statements that were shown on screen and spoken by audio file in the same female voice as the intervention (e.g., “I keep having worrying thoughts that won’t leave my mind”). The statements were randomly selected from a list of 16 statements with each statement being presented twice. These mood induction statements have been shown to induce negative mood and augment drug choice and craving (Hogarth & Hardy, 2018). Participants were instructed to read each statement and imagine moving into that state. While watching the negative mood induction video, participants in the breath counting condition were asked to deploy the breath counting technique.

#### Measures

Following questions of age, gender, native English, and frequency of drinking, participants completed the following questionnaires: (1) AUDIT which contained 10 items assessing alcohol use and problems in the past 12 months. Total scores range from 0 to 40 split into low-risk (0–7), hazardous (8–15), harmful (16–19), and possibly dependent (20–40) categories. (2) The adult Patient-Reported Outcomes Measurement Information System Alcohol Use Short Form (PROMIS; Pilkonis et al., 2016) contained 7 items assessing loss of control over drinking in the past 30 days, endorsed on a 1–5 scale ranging from *Never* to *Always* (average scale scores are reported). (3) The modified 5-factor Drinking Motives Questionnaire Revised (DMQR; Grant et al., 2007) measured how frequently drinking is motivated by each reason listed, on a 1–10 scale ranging from *Never* to *Almost always*. It has 5 subscales: drinking for conformity, enhancement, socialising, drinking to cope with depression, and drinking to cope with anxiety. These latter 2 subscales (drinking to cope with depression and drinking to cope with anxiety) were averaged since they correlated so highly (*r* = 0.84, *p* < 0.001). (4) The Generalised Anxiety Disorder scale (GAD; Spitzer et al., 2006) contained 7 items assessing GAD symptoms in the past 2 weeks (e.g., “feeling nervous, anxious or on edge”) from 0 (*Not at all*) to 3 (*Nearly every day*). The total sum score ranges from 0 to 21, with scores of 5, 10, and 15 as the cut-off points for mild, moderate, and severe GAD. (5) The Patient Health Questionnaire depression scale (PHQ; Kroenke et al., 2009) contained 8 items assessing depressive symptoms in the past 2 weeks (e.g., “little interest or pleasure in doing things”) from 0 (*Not at all*) to 3 (*Nearly every day*). Total sum scores range from 0 to 24, with scores of 5, 10, 15, and 20 representing cut-off points for mild, moderate, moderately severe, and severe depression, respectively. Regarding reliability, the values for McDonald’s omega across the three studies indicated an acceptable to good reliability for the measures used (Table 1).

**Table 1:** Characteristics (mean, standard deviation, range) of participants in the breath counting and control intervention group in Study 1 (A), Study 2 (B), and Study 3 (C)

	(A) Study 1				(B) Study 2				(C) Study 3			
	Breath counting (*n* = 68)	Control (*n* = 54)	*p*	*ω*	Breath counting (*n* = 61)	Control (*n* = 50)	*p*	*ω*	Breath counting (*n* = 32)	Control (*n* = 30)	*p*	*ω*
Age	31.87, 8.92, 19–52	30.61, 8.72, 18–49	0.43		20.48, 2.43, 18–32	20.36, 2.12, 18–28	0.79		26.94, 9.10, 19–57	29.87, 12.07, 18–66	0.28	
Gender ratio (m/f)	38/30	26/28	0.33		11/50	11/39	0.60		24/8	17/13	0.18	
Alcohol units	-	-	-		3.53, 1.60, 1.15–4.50	4.23, 4.58, 1–18.20	0.75		3.7, 2.90, 0–9.2	3.3, 2.80, 0–9.2	0.60	
AUDIT	25.61, 9.30, 8–40	26.64, 8.16, 9–40	0.11	0.91	14.41, 5.10, 8–25	15.04, 5.74, 8–29	0.54	0.74	18.21, 7.08, 9–40	15.9, 4.61, 9–27	0.13	0.71
PROMIS	3.28, 0.94, 1–5	3.59, 0.74, 1.14–4.86	0.04	0.92	2.44, 0.74, 1–4.43	2.29, 0.69, 1–3.86	0.28	0.85	2.66, 0.83, 1–4.71	2.8, 0.70, 1.57–4.43	0.48	0.85
DMQR total score	6.81, 2.07, 1.89–10	7.26,1.62, 3.89–9.5	0.85	0.97	4.47,1.56, 1.75–7.96	4.49, 1.34, 1.64–7,11	0.92	0.91	4.0, 1.48, 1.39–6.67	4.19,1.90, 1.57–8.46	0.66	0.71
DMQR coping	6.69, 2.32, 0.72–10	7.28, 1.76, 2.65–10	0.18	0.94	3.91, 2.10, 0–8.64	3.87, 1.92, .50–7.40	0.93	0.92	4.19, 1.90, 1.57–8.46	3.82, 2.52, 0.76–9.10	0.76	0.94
DMQR enhancement	7.21, 1.85, 3.20–10	7.58, 1.85, 1.80–10	0.12	0.87	4.87, 1.82, 0–8.20	5.44, 1.60, 1.20–8.60	0.08	0.77	5.0, 1.70, 2.2–8.2	5.4, 2.20, 1.2–8.6	0.42	0.75
DMQR socialising	7.54, 1.61, 2.60–10	7.89,1.48,3.80–9.80	0.27	0.83	6.47, 1.40, 2–8.80	6.46, 1.22, 3.40–8.40	0.96	0.68	6.41, 1.74, 2.2–9.6	7.1, 1.50, 3.8–9.2	0.11	0.68
DMQR conformity	6.22, 2.78, 0–10	6.48, 2.52, 0–9.40	0.22	0.93	2.13, 1.95, 0–7.40	1.94, 1.99, 0–6.20	0.61	0.87	1.01, 1.42, 0–5.80	1.6, 2.30, 0–6.8	0.26	0.93
GAD-7 Anxiety	7.66, 5.08, 0–19	7.40, 5.17, 0–19	0.78	0.90	7.90, 5.29, 0–21	7.36, 5.29, 0–21	0.59	0.89	7.71, 6.30, 0–21	6.9, 5.40, 0–21	0.60	0.89
PQH-8 Depression	8.64, 5.60, 0–21	8.81, 5.85, 0–18	0.87	0.90	9.75, 5.43, 0–22	7.94, 4.5, 0–19	0.06	0.85	9.06, 6.85, 0–22	6.4, 5.30, 0–24	0.09	0.86

Each characteristic was entered into a *t*-test comparing the two groups (gender ratio was tested with a chi-square), and column *p* reports the significance level. There were no significant group differences. *AUDIT*, Alcohol Use Disorders Identification Test; *PROMIS*, Patient-Reported Outcomes Measurement

Baseline craving was measured with the Alcohol Craving Questionnaire–Short Form (ACQ) which contained 12 items (e.g., “If I had some alcohol, I would probably drink it”) endorsed on a 1–7 scale from *Strongly Disagree* to *Strongly Agree* (Tiffany & Drobes, 1991). Participants then rated their baseline subjective happiness and sadness on a 1–5 scale ranging from *Not at all* to *Very*.

Following mood induction, alcohol craving and subjective mood (happiness/sadness) were measured again in the same way as at baseline. Breath counting training was expected to attenuate the mood-induced change in these scores.

#### Data Analyses

The data met assumptions for ANOVA in most cases, and where not, there were only minor violations, which ANOVA is generally regarded as robust against. A 2 × 2 mixed model ANOVA was conducted for each of the three outcome measures (alcohol craving, subjective happiness, subjective sadness) with the between-subjects variable intervention group (breath counting, control) and the within-subjects variable timepoint (baseline, post mood induction). Significant interactions were followed up by one-way ANOVAs testing the group effect at each level of timepoint and the timepoint effect at each level of group.

### Results

**Fig. 1:**
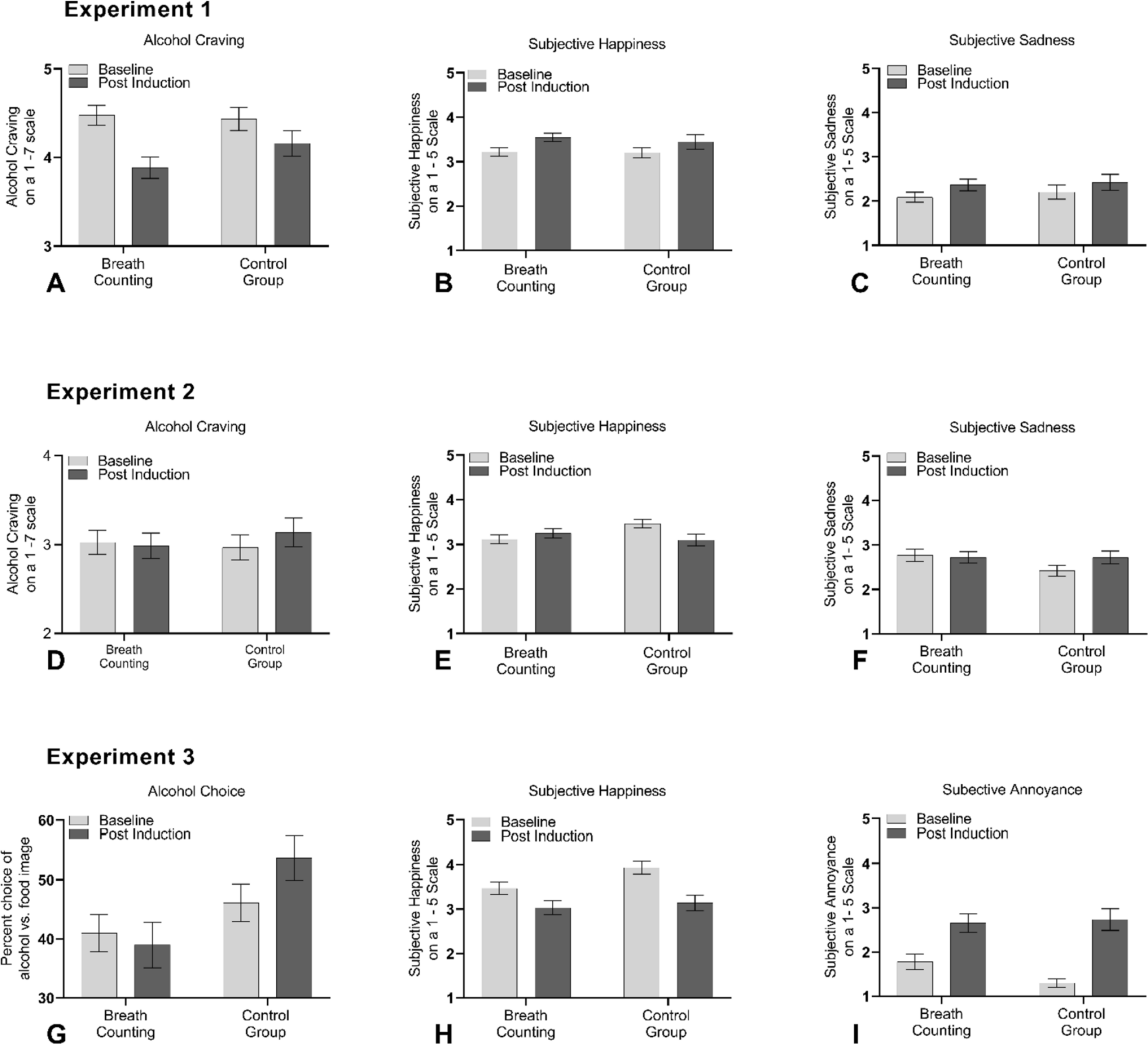
**A**–**I** Measures of alcohol motivation (alcohol craving, alcohol choice) and subjective mood recorded at baseline and again following the intervention (breath counting or control) plus mood induction procedure

As shown in Table 1A, the breath counting and control group did not significantly differ with respect to questionnaire characteristics. Analysis of the craving data shown in Fig. 1A yielded a non-significant main effect of group, a significant main effect of timepoint, and a significant interaction between group and timepoint, *F*(1,120) = 3.93, *p* = 0.04, *η*_*p*_^2^ = 0.03. Breakdown of this interaction revealed a significant main effect of timepoint, *F*(1, 120) = 30.18, *p* < 0.01, *η*_*p*_^2^ = 0.20, in both the breath counting, *F*(1,67) = 34.67, *p* < 0.001, *η*_*p*_^2^ = 0.34, and the control group, *F*(1,53) = 4.96, *p* = 0.03, *η*_*p*_^2^ = 0.08, with the two groups differing at the post induction timepoint, *F*(1,121) = 2.13,* p* = 0.147, but not at baseline, *F*(1,121) = 0.05, *p* = 0.816. These results suggest that the mood induction manipulation was ineffective in increasing craving. Rather, craving decreased over time or with repeated testing, and this decrease was more substantial for the breath counting than control group, providing preliminary support for an effect of breath counting in reducing alcohol craving generally. However, whether breath counting attenuates mood-induced craving specifically cannot be addressed, because there was no mood induction effect on craving.

Analysis of the subjective happiness data showed no significant main effect of group, *F*(1,120) = 0.50, *p* = 0.47,* η*_*p*_^2^ = 0.004, or timepoint, *F*(1,120) = 2.43, *p* = 0.121, *η*_*p*_^2^ = 0.02, or interaction between group and timepoint, *F*(1,120) = 0.16, *p* = 0.68,* η*_*p*_^2^ = 0.00. Similarly, analysis of the subjective sadness data revealed no significant main effect of group, *F*(1,120) = 0.25, *p* = 0.61,* η*_*p*_^2^ = 0.00, or interaction between group and timepoint *F*(1,120) = 0.07, *p* = 0.78, *η*_*p*_^2^ = 0.00. There was, however, a significant main effect of timepoint, *F*(1,120) = 5.57, *p* = 0*.*02, *η*_*p*_^2^ = 0.04, suggesting that subjective sadness increased over time across the sample.

### Discussion

Study 1 found that, in a fully online design, deployment of a briefly trained breath counting technique produced a larger decrease in alcohol craving than a control intervention, suggesting breath counting has some efficacy in reducing craving. However, the negative mood induction procedure failed to produce the expected increase in alcohol craving or worsening of subjective mood, so the study could not assess the impact of breath counting on mood-induced craving specifically.

## Study 2

### Method

#### Participants

The inclusion and exclusion criteria were identical to Study 1 and the sample of Study 2 was fully independent from Study 1. One hundred sixty-three participants met the inclusion criteria of which 42 were excluded for outlying long survey times, and 6 were excluded for drug use on the day of the experiment. In addition, it was noted that 4 participants showed extreme change in their alcohol craving or subjective mood from baseline to testing (greater than 3 times the interquartile range), so they were excluded on the assumption that they were not attending to the task. This left 111 participants (20% male) in the final analysis with 61 in the breath counting and 50 in the control group. Although our final sample size was reduced from 163, the final sample size remains powerful at > 99% to detect a negative mood induction effect.

Participants who completed the study received a £5 reimbursement via direct bank transfer. This method was selected as an alternative to the Amazon voucher used in Study 1 to discourage multiple responses and fraudulent participants as each response would require a unique bank account (Teitcher et al., 2015). Participants provided informed signed consent. The study was approved by the School of Psychology Research Ethics Committee.

#### Procedure

Study 2 used the same procedure as Study 1 except that the negative mood induction protocol was strengthened by including 32 unique depressive-ruminative statements rather than 16 statements repeated twice in the hope of increasing the magnitude of the mood induction effect (which was absent in Study 1). As in Study 1, participants were randomly assigned to the breath counting or control groups using simple random sampling on a 1:1 ratio. The mood induction video remained at 3.5 min long.

#### Measures

Alcohol craving and subjective mood were measured as in Study 1.

#### Data Analyses

The data analyses were the same as Study 1.

### Results

As shown in Table 1B, the breath counting and the control group in Study 2 did not significantly differ with respect to questionnaire characteristics. Analysis of the craving data, shown in Fig. 1D, showed no significant main effects of group, *F*(1,109) = 0.05, *p* = 0.81, *η*_*p*_^2^ = 0.00, or timepoint, *F*(1,109) = 1.74, *p* = 0.18, *η*_*p*_^2^ = 0.01, but yielded a significant interaction between group and timepoint, *F*(1,109) = 4.46, *p* = 0.03, *η*_*p*_^2^ = 0.03. Breakdown of this interaction indicated that there was a significant increase in craving from baseline to the post mood induction timepoint in the control group *F*(1,49) = 5.40, *p* = 0*.*02, *η*_*p*_^2^ = 0.09, but not in the breath counting group,* F*(1,60) = 0.34, *p* = 0*.*559, *η*_*p*_^2^ = 0.00. However, the two groups did not differ significantly at baseline, *F*(1,110) = 0.90, *p* = 0*.*763, *η*_*p*_^2^ = 0.00, or post mood induction *F*(1,110) = 0.48, *p* = 0*.*49, *η*_*p*_^2^ = 0.00. These findings confirmed Shuai et al. (2020) in showing that breath counting can attenuate a negative mood–induced increase in alcohol motivation, but the current study showed this effect in hazardous community drinkers, with the intervention and testing protocol delivered online.

Analysis of subjective happiness data yielded no significant main effect of group, *F*(1,109) = 0.53, *p* = 0.46, *η*_*p*_^2^ = 0.00, or timepoint, *F*(1,109) = 2.88, *p* = 0.09, *η*_*p*_^2^ = 0.02, but revealed a significant interaction between group and timepoint, *F*(1,109) = 13.26*, p* < 0.001, *η*_*p*_^2^ = 0.10, driven by a significant decrease in happiness from baseline to post mood induction in the control group, *F*(1,49) = 11.53, *p* < 0.001, *η*_*p*_^2^ = 0.19, which was not significant in the breath counting group,* F*(1,60) = 2.33, *p* = 0.13,* η*_*p*_^2^ = 0.03. However, interpretation of this difference is complicated by the observation that the control group reported greater happiness than the breath counting group at baseline, *F*(1,110) = 6.30, *p* = 0.01, *η*_*p*_^2^ = 0.05, but not post mood induction timepoint,* F*(1,110) = 0.77, *p* = 0.37, *η*_*p*_^2^ = 0.00, suggesting that the differential change in happiness between groups could reflect the effects of breath counting on mood induction–induced decrease in happiness, or this effect may simply reflect regression to the mean.

Analysis of the sadness data revealed no significant main effects of group, *F*(1,109) = 1.00, *p* = 0.31, *η*_*p*_^2^ = 0.00, or timepoint, *F*(1,109) = 2.79, *p* = 0.09, *η*_*p*_^2^ = 0.02, but a significant group by timepoint interaction, *F*(1,109) = 5.42, *p* = 0.02, *η*_*p*_^2^ = 0.04, driven by an increase in sadness from baseline to the post mood induction timepoint in the control group, *F*(1,49) = 7.23, *p* = 0*.*01, *η*_*p*_^2^ = 0.12, but not the breath counting group,* F*(1,60) = 0.24, *p* = 0.62, *η*_*p*_^2^ = 0.004. However, similar to happiness data, the groups differed marginally at baseline, *F*(1,109) = 3.44, *p* = 0.06, *η*_*p*_^2^ = 0.03, and non-significantly at the post mood induction timepoint,* F*(1,110) = 0.00, *p* = 0.99,* η*_*p*_^2^ = 0.00, consistent with either the effects of breath counting, or this effect may simply reflect regression to the mean.

### Discussion

Study 2 used a recruitment plan designed to improve online data quality (primarily by offering reimbursement via bank transfer rather than Amazon vouchers), and employed a more compelling negative mood induction procedure (with more unique statements designed to elicit feelings of sadness). In this study, mood induction was effective in increasing craving and worsening subjective mood. Most importantly, deployment of the breath counting technique eliminated the negative mood–induced increase in alcohol craving, demonstrating our target effect. Breath counting also appeared to abolish the mood-induced worsening of subjective mood, but because groups differed at baseline, this effect may have simply reflected regression to the mean.

## Study 3

### Method

#### Participants

Study 3 recruited 62 drinkers (66% male) in pubs in Exeter city. Participants were included if they reported an AUDIT score greater than 8 indicating hazardous drinking and self-reported being “not at all” intoxicated, but not if they reported being “mildly” or “very intoxicated” to minimise any stress damping effect of alcohol (Balodis et al., 2011; Wilkie & Stewart, 2005) and to avoid ethical concerns. Two participants were excluded for reporting drinking an outlying (greater than 1.5 times the interquartile range) number of alcohol units despite claiming to be “not at all intoxicated”. The study was approved by the University of Exeter Psychology Research Ethics Committee. Participants were paid £5 for participation.

A statistical power analysis was performed for sample size estimation. The effect size (ES) in this study was considered to be medium using Cohen’s (1988) criteria. With an alpha = 0.05 and power = 0.95, the projected sample size needed with this effect size (GPower 3.1) is approximately *n* = 54. Thus, our proposed sample size of *n* = 62 was adequate for the main objective of this study.

#### Procedure

After obtaining permission from local pub managers, the study researchers presented in local pubs during the day (from 12 to 7 pm). For safety reasons, the study researchers would usually present in pairs; however, participant recruitment was usually led by one of the two researchers. The researcher would approach drinkers and introduced themselves and the purposes of the study. Breath alcohol content (BrAC) was omitted as a measure due to concerns about extra burden on participants’ and disruption of the pub environment, meaning pub managers would not allow access to researchers. After obtaining written informed consent, the researcher would invite the participant to an individual table with the laptop facing the wall to preserve privacy. Participants were randomized using simple random sampling on a 1:1 ratio as in Studies 1 and 2.

#### Measures

Paper questionnaires assessing participant characteristics at the outset of the study were identical to Studies 1 and 2. With respect to outcome measures, alcohol motivation was measured with an alcohol choice computer task rather than subjective craving because we have used this measure extensively to detect mood induction effects (Hogarth & Field, 2020) and the protective effect of breath counting (Shuai et al., 2020), and because this measure has greater translation to animal models of drug motivation (Banks & Negus, 2017). Baseline alcohol choice was measured in 24 two-alternative pictorial choice trials. In each trial, participants freely chose to enlarge either an alcohol or food thumbnail image, presented randomly in the left and right side of the screen, by pressing the left or right arrow key (Shuai et al., 2020). The alcohol and food stimuli were sampled from a set of 28 images each. The dependent variable was the percentage choice of alcohol pictures across all choice trials.

Subjective mood was measured at baseline by asking participants to what extent they currently felt “happy” and “annoyed”, in random order, on a 5-point scale ranging from 1 (*not at all*) to 5 (*extremely*). We switched from sadness to annoyance because participant feedback from previous noise stress induction studies indicated that the noise was annoying rather than stressful.

Participants were then randomly assigned to the breath counting or control group, after which participants completed the alcohol pictorial choice task but with the addition of a loud and unpleasant industrial noise stressor (70 dB), played simultaneously through headphones over 36 trials. This stressor was expected to augment alcohol choice (Shuai et al., 2020). The breath counting group were instructed to deploy the breath counting technique during the stress test, whereas the control group received no instruction. Finally, participants reported their subjective happiness and annoyance.

#### Data Analyses

The data analysis was the same as in Studies 1 and 2.

### Results

As shown in Table 1C, the breath counting and control group in Study 3 did not significantly differ with respect to questionnaire characteristics. Analysis of the alcohol choice data shown in Fig. 1G yielded no significant main effect of timepoint, *F*(1,60) = 2.39, *p* = 0.12, *η*_*p*_^2^ = 0.03, but a significant main effect of group, *F*(1,60) = 4.54, *p* = 0.03, *η*_*p*_^2^ = 0.07, qualified by a significant interaction between group and timepoint, *F*(1,60) = 7.20, *p* = 0.009, *η*_*p*_^2^ = 0.10. This interaction was driven by an increase in alcohol choice between baseline and the post induction timepoint in the control group, *F*(1,29) = 7.24, *p* = 0.01, *η*_*p*_^2^ = 0.20, which was eliminated in the breath counting group, *F*(1,31) = 0.82, *p* = 0.37,*η*_*p*_^2^ = 0.02. Furthermore, the two groups differed significantly in the post induction timepoint, *F*(1,60) = 7.32, *p* = 0.009,*η*_*p*_^2^ = 0.10, but not at baseline, *F*(1,60) = 1.29, *p* = 0.26,*η*_*p*_^2^ = 0.02. These findings confirm that breath counting attenuates mood-induced alcohol motivation, as seen in Shuai et al. (2020) and Study 2. In the current study, this effect was found in hazardous community drinkers in a pub context, supporting the utility of the breath counting training in a natural high-risk environment.

Analysis of the subjective happiness data revealed a non-significant main effect of group, *F*(1,60) = 2.79, *p* = 0*.*10, *η*_*p*_^2^ = 0.04, and interaction between group and timepoint, *F*(1,60) = 1.63, *p* = 0.20, *η*_*p*_^2^ = 0.02. However, there was a significant main effect of timepoint, *F*(1,60) = 25.60, *p* < 0.001, *η*_*p*_^2^ = 0.29, indicating that happiness decreased after negative mood induction and breath counting did not influence this effect. Similarly, analysis of the subjective annoyance data revealed no significant main effect of group, *F*(1,60) = 0.94, *p* = 0.33, *η*_*p*_^2^ = 0.01, or interaction between group and timepoint, *F*(1,60) = 2.56, *p* = 0.11, *η*_*p*_^2^ = 0.04. However, there was a significant main effect of timepoint, *F*(1,60) = 43.85, *p* < 0.001, *η*_*p*_^2^ = 0.42, indicating that mood induction increased annoyance. However, breath counting did not influence this effect significantly.

### Discussion

In Study 3, breath counting eliminated a noise-stress-induced increase in alcohol choice in hazardous community daytime drinkers tested in a pub context, confirming the effects of breath counting in the natural environment. Breath counting did not have any effects on mood-induced worsening of subjective mood (happiness, annoyance), supporting the conclusion that breath counting does not reliably produce this effect. However, breath counting produced a protective effect against the mood-induced changes in these outcome measures (alcohol choice) as revealed by a significant group by timepoint interaction. This significant interaction was found for all measures of alcohol motivation across the three studies shown in Fig. 1A, D, and G, suggesting breath counting attenuated alcohol motivation overall (A) and the mood-induced increase in alcohol motivation (D and G). However, there was no protective effect of breath counting on the mood-induced worsening of subjective mood (Fig. 1B, C, E, F, H, I). These findings demonstrate the reliability of the protective effect of breath counting in hazardous drinkers, across different procedures.

## General Discussion

Overall, the effects of breath counting in attenuating negative mood–induced craving and abolishing stress-induced increases in alcohol choice were consistent across the three studies. However, the effects of breath counting on the mood-induced worsening of subjective mood (happiness, sadness, and annoyance) were null in Studies 1 and 3, and uninterpretable in Study 2 due to group differences at baseline. The reliable finding in all three studies was that breath counting attenuates mood-induced alcohol motivation, corroborated a study with student drinkers (Shuai et al., 2020). However, whereas this study suggested that hazardous drinkers were less sensitive to the effects of breath counting on mood-induced alcohol motivation, the three studies reported here indicate that breath counting does exert this effect in hazardous drinkers in a real-world context. This effect can be achieved with online delivery of the breath counting training, and operates within natural high-risk drinking environments.

However, it remains unclear by what mechanism breath counting attenuated stress-induced alcohol choice and craving in the current studies. The finding that the breath counting training (versus control) did not reliably attenuate worsening of subjective mood indicates that this effect could not have mediated the effect on alcohol motivation. One possible therapeutic mechanism is that breath counting distracted attention from the mood statements and noise stressors used to induce negative mood in these studies (Tapper, 2018). Indeed, studies have shown that attention-demanding tasks, where attention is directed to exteroceptive stimuli, can attenuate craving (Dodds et al., 2019; May et al., 2010). More recently, we tested this possibility by including active control participants who participated in a simple distraction task (Herchenroeder et al., 2023). In this study, breath counting produced comparable effects to simple distraction in terms of attenuating stress-induced alcohol choice suggesting that the therapeutic effects of breath counting may stem from distraction.

### Limitations and Future Directions

The three studies have several limitations. A primary limitation of our study is its modest sample sizes which limits the generalizability of our findings. The online design expedited recruitment of hazardous drinkers, but the sample is likely to have contained a preponderance of people who regularly participate in online surveys (Chandler et al., 2014) and even fraudulent responders (Chandler & Paolacci, 2017). This also explains the small effect sizes observed in online Studies 1 and 2, which is typically expected for online studies. The additional screening methods used mitigated the effect of low-quality data, but also reduced power (Buchanan & Scofield, 2018) and raises the risk of false positives due to the additional degrees of freedom in the analysis (Simmons et al., 2011). The risk of false positives, however, was mitigated by the replication of the effect in multiple studies, but ideally, in future work, exclusion criteria would be pre-registered following a standard protocol guided by the current approach (Teitcher et al., 2015). In addition, we did not collect any demographic information on the racial and ethnic composition of our samples. Secondly, our study is also limited by the use of self-report measures for our primary outcomes (alcohol cravings, self-report mood) in Studies 1 and 2 and the lack of objective verification in all three studies. Subjective responses are prone to demand characteristics, where participants report what they consider is expected of them (McCambridge et al., 2012). In addition, we could not isolate any expectancy effects in this study that may have potentially influenced any responses in the breath counting group. Our studies did not contain any physiological measures of assessing the breathing rate of study participants, neurophysiological measures for stress reactivity or state mindfulness, or any objective measures to determine the participants’ level of intoxication (BrAC) due to practical constrains. Consequently, our work is limited in its specificity of potential neurophysiological mechanisms involved. Future studies could include physiological measures to quantify compliance with the breath counting instructions (Levinson et al., 2014) or protection from physiological stress (Garland et al., 2010; Paz et al., 2017) to determine if these effects mediate or moderate the effects of breath counting on mood-induced alcohol motivation as well as self-report measures to quantify change in state mindfulness after breath counting training (Bravo et al., 2018). Ultimately, it is important to isolate the therapeutic mechanism so that this can be optimised within the intervention protocol (Morgenstern & McKay, 2007). A third limitation is the weakness of the negative mood induction procedure that was developed for Studies 1 and 2. Previous studies have shown that mindfulness training may successfully attenuate stress reactivity and support the psychophysiological recovery from alcohol (Garland et al., 2010) and drug-related cues (Garland et al., 2019). Given the online design of our studies (Studies 1 and 2), our study was constrained in terms of stress and cue manipulations and we opted for a negative mood induction procedure that used verbal prompts and instead produce nil (Study 1) or very mild (Study 2) effects. The ecological validity of this mood induction procedure is unknown, although the verbal prompts hold face-validity in terms of their apparent similarity to real-world experience. Future studies could further investigate the effects of breath counting training on alcohol motivation using more robust negative mood induction methods such as personalised stress narratives and alcohol cue exposure. And last, a limitation of our work is that Studies 1 and 2 took place online and Study 3 took place in a pub, which likely increased the impact of extraneous variables on the data quality such as external distractions (Clifford & Jerit, 2014). However, the demonstration of effects of breath counting on alcohol motivation despite such variation in environment supports the ecological validity of the brief breath counting training.

Some strengths of the studies were the demonstration of a comparable effect across studies despite the use of difference outcome measures (alcohol picture choice and alcohol craving) and two different negative induction protocols (online depressive-ruminative statements and noise stress), supporting the generality of the effects. The briefness of the training protocol, and its exclusive focus on breath counting, means we cannot claim comparability with full mindfulness packages, but the similar findings obtained with the brief and full intervention packages support their comparability. Finally, the briefness of the training protocol, and its availability online (https://www.youtube.com/watch?v=tnFUvLIBhKQ) as well as the demonstrated efficacy with online delivery, and in the natural environment, increases the attractiveness of this brief intervention, although future studies are necessary to further explore the clinical potential and utility of this brief breath counting training as a stand-alone or homework component to larger therapeutic packages.

To conclude, the three studies found that brief training and deployment of a breath counting technique, compared to a control group, reduced alcohol craving (Study 1) and eliminated a negative mood–induced increase in alcohol craving and choice (Studies 2 and 3) in hazardous community drinkers, with these effects operating online (Studies 1 and 2) and within a pub environment (Study 3). Similar effects have been obtained with full mindfulness packages (Bowen et al., 2014; Stasiewicz et al., 2018), suggesting that mindfulness training may improve drinking outcomes by building resilience to negative affect drinking triggers. The therapeutic effect seen in hazardous drinkers, within a pub and online, with different negative affect induction protocols, and different measures of alcohol motivation, and the briefness of the training protocol and its online availability all increase the attractiveness of this approach and expand its potential applications in the treatment of substance use problems. There remains an empirical question as to why breath counting attenuates negative mood–induced alcohol motivation which should be identified to better tailor the intervention method.

## Data Availability

All data and study materials are available at the Open Science Framework 10.17605/OSF.IO/D7KPT.

## Declarations

### Artificial Intelligence

During the preparation of this work, no artificial intelligence tools/services were used.
